# High-resolution reconstruction of the United States human population distribution, 1790 to 2010

**DOI:** 10.1038/sdata.2018.67

**Published:** 2018-04-24

**Authors:** Yu Fang, James W. Jawitz

**Affiliations:** 1 Soil and Water Sciences Department, University of Florida, Gainesville, Florida 32611, USA

**Keywords:** Geography, Environmental chemistry

## Abstract

Where do people live, and how has this changed over timescales of centuries? High-resolution spatial information on historical human population distribution is of great significance to understand human-environment interactions and their temporal dynamics. However, the complex relationship between population distribution and various influencing factors coupled with limited data availability make it a challenge to reconstruct human population distribution over timescales of centuries. This study generated 1-km decadal population maps for the conterminous US from 1790 to 2010 using parsimonious models based on natural suitability, socioeconomic desirability, and inhabitability. Five models of increasing complexity were evaluated. The models were validated with census tract and county subdivision population data in 2000 and were applied to generate five sets of 22 historical population maps from 1790–2010. Separating urban and rural areas and excluding non-inhabitable areas were the most important factors for improving the overall accuracy. The generated gridded population datasets and the production and validation methods are described here.

## Background & Summary

Human actions have caused substantial alterations to the Earth, transforming the landscape, affecting ecosystem patterns and processes, driving biodiversity loss, altering global hydrological and biogeochemical cycles, amplifying resource exploitation and environmental deterioration, and contributing to climate change^1–3^. Such ecological and societal consequences vary across space. Human population density is considered to be a useful indicator of the type and intensity of the human environment interaction, with higher population density leading to higher levels of impacts^4–7^. Therefore, it is vital to create reliable spatially explicit, high-resolution estimates of the human population distribution to advance our understanding of coupled human and natural systems, to provide support to policy decision-making, and to achieve ecological and socioeconomic sustainability^8^.

Census data have been routinely collected and applied, however, such data are ascribed to defined administrative units, leading to abrupt changes in population at the administrative boundary, and masking of spatial heterogeneity within administrative units^9,10^. For the conterminous US, census data are available at the county level from 1790 to 2010 (ref. 11). These data could provide strong support for improved understanding of human-environment interactions, if more refinement was possible within the administrative units. Dasymetric mapping is an areal interpolation to disaggregate data from a set of areal units into finer units using ancillary data^12^. The modern revolution in geospatial data availability greatly facilitates dasymetric mapping and the creation of more accurate data on population distribution^13^, including the Gridded Population of the World (GPW)^14^, the Global Rural Urban Mapping Project (GRUMP)^15^, LandScan^16^, and WorldPop^17^.

A recent challenge has been to map the human population distribution in low income nations, where detailed updated census data or high resolution geospatial data are lacking^18–21^. Similar challenges from insufficient data availability occur in estimating historical population distributions. The high resolution mapping efforts described above were all developed based on modern census data since 1990, and are of limited utility for long-term (e.g., multi-decadal) dynamic analyses^22,23^. A notable exception is the History Database of the Global Environment (HYDE)^24^. While the temporal range from HYDE is vast (from 1000 BC to 2005 AD), the 10-km spatial resolution is relatively low, constrained by input census data.

Major influencing factors that have been considered in human population mapping include land cover, night lights, topography, urban areas, and roads^25–27^. Land use/land cover data, derived from remote sensing images, best reflect population density and have been suggested as an important source for human population mapping^19,28^. However, such imagery data only became available beginning with the launch of the first land satellite, Landsat-1, in 1972. Several global land cover datasets^29–33^ have used census data as an input or a proxy for human activities to simulate the spatial map of anthropogenically managed land (e.g. cropland, pasture). However, it would be circular and infeasible to use such derived land cover data to model population distribution.

The goal of this study is to generate spatially explicit human population distribution maps for the conterminous US that could be used to advance studies of anthropogenic effects on the environment, and to provide support for policy decision-making. Recognizing that the spatial resolution of census administrative boundaries is the principal factor affecting map accuracy^21^, we use county-level census data, as this is the highest resolution data consistently available from 1790 to 2010. Despite the scarcity of reliable historical land cover maps, the separation of urban and rural settlement areas could significantly improve the accuracy of population distribution mapping^19^, since 80.7% of the US population lived in urban areas, which covered only about 3.1% of the total land area, according to the 2010 US census^34^. Power-law scaling relationships^35^ between urban area and population were applied to estimate historical urban areas. Additional data including elevation, water bodies, and protected areas were used to allocate population to urban and rural areas within counties. Five models of increasing complexity, from one (M1) to eight variables (M5), were developed to model population distribution. The models were validated with measured data from 2000 based on comparison with census data at the tract and county subdivision levels, and applied to generate five sets of 22 historical decadal population maps from 1790–2010 (Historical population dataset for the conterminous US, Data Citation 1). The model-generated urban extents were also assessed in two fast-growing regions in which historical data were available^36,37^: San Francisco/Sacramento and Baltimore/Washington DC.

## Methods

### Data collection

**Figure 1:**
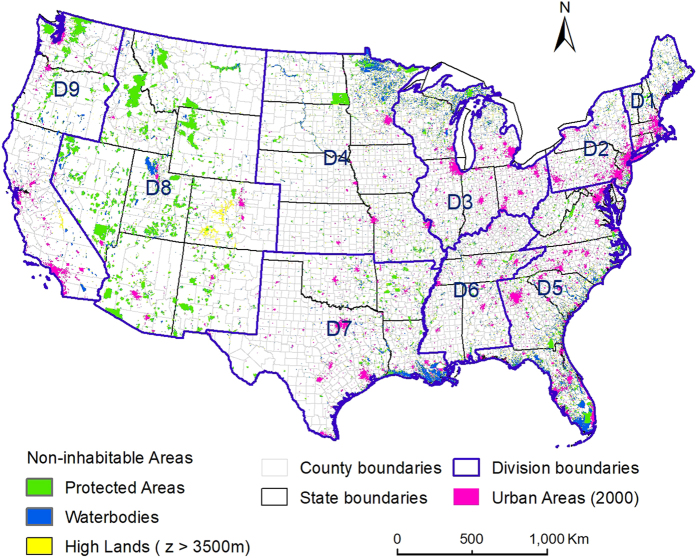
The major spatial input data for the population downscaling models, and associated administrative unit boundaries (census divisions D1 to D9, state, county, and urban areas). Un-inhabitable areas include designated protected areas, waterbodies, and highlands with eleviation, z, larger than 3500 m.

Census data at four levels of spatial resolution, mainly obtained from the National Historical Geographic Information System (NHGIS) (https://www.nhgis.org/)^38^, were used: total and urban population at county level (1790–2010), county subdivision population (1980–2010), census tract population (1990–2010), and population for urban areas (2000 and 2010). Note that American Indians were not fully included in the census prior to 1900 (ref. 39). Our results thus underestimated populations where American Indians settled. County, county subdivision, census tract, and urban area boundary shapefiles were also obtained from NHGIS. Census division boundaries and population for selected urban areas in earlier decades were obtained from the US Census Bureau (Fig. 1).

**Figure 2:**
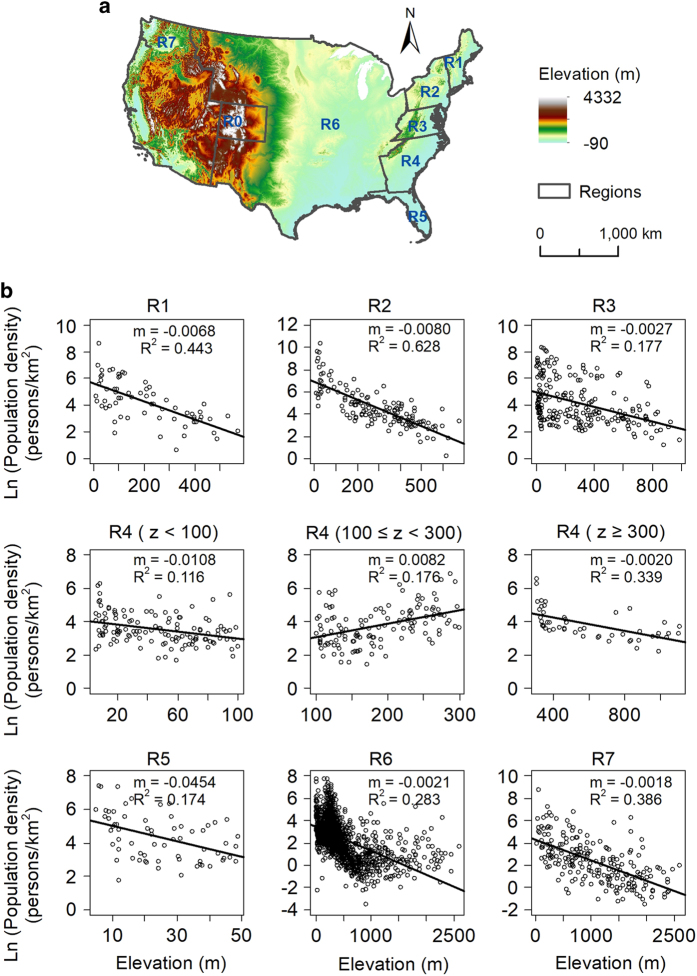
Topo-demography relationships. Topography and boundaries for regions R0 to R7 for the conterminous US (**a**), and relationships between log population density and county mean elevation in 2000 for the seven regions (**b**). Region 4 is divided into three sub-regions by elevation. Solid lines are linear regressions with slope m.

Waterbody data (medium resolution, at 1:100 000-scale), including lake/pond features, swamps/marshes, reservoirs, playas, estuaries, and ice mass, were completed in 2001 and derived from the National Hydrography Dataset (http://nhd.usgs.gov/). Protected areas data (version 1.3), designated to preserve biological diversity and other natural, recreation and cultural uses, were released in 2012 and obtained from National Gap Analysis Program (http://gapanalysis.usgs.gov/padus/). Digital elevation model data (Fig. 2) were released in 2013 and derived from NASA Shuttle Radar Topography Mission Version 3.0 (http://www2.jpl.nasa.gov/srtm/), with a resolution of 90 m. Historical data on urban spatial extents for San Francisco/Sacramento and Baltimore/Washington DC region^36,37^ were from the USGS Land Cover Institute (LCI).

### Level of spatial units

The above data were used to compute population for each inhabited pixel (*k*) in each decade (*t*) from 1790 to 2010 (excluding 1960). The data were variously applied over the following spatial units: urban area (*φ*), census tract (*c*_1_), county subdivision (*c*_2_), county (*i*), census division (*δ*), and Region (*μ*). There were 9 census divisions (*δ*: D1 – D9) and 7 Regions (*μ*: R1 – R7) with constant boundaries over time, while the number of other spatial units varied with the expansion of human settlements and the modification of administrative boundaries. For example, the number of counties increased from 292 in 1790 to 3109 in 2010. Also, in 2010, there were 7,754 146 pixels (1 km^2^), 72,271 census tracts, 35,532 county subdivisions, and 3,535 urban areas in the conterminous US.

### Population count determination

Decennial census data on total population (*P*_*T*_) and aggregated urban population (*P*_*U*_) for each urban area (*φ*) determined the remaining rural population (*P*_*R*_) by difference, for each county (*i*) from 1790 to 2010 (excluding 1960, for which digital urban population data are missing):

(1)PR=PT−∑PU,φ

The number of counties containing urban population increased from 19 in 1790 to 2424 in 2010. For counties with more than one urban area, we obtained the population for individual urban areas from the US Census Bureau. Note a population threshold of 2500 was used for identifying urban areas (https://www.census.gov/geo/reference/ua/urban-rural-2010.html).

### Areal extent delineation

The 2000 census includes full coverage of areal extent (*A*_*U*_) for all of the 3610 urban areas. However, areal extents are incomplete for 1990 and largely unknown in prior decades. The 1990 NHGIS data cover only 396 large urban areas with population larger than 50,000. Thus, the 2000 data were used as a baseline for historical backward projections of urban areal extents. The areal extent of each urban area (*φ*) in a census division (*δ*) prior to 2000 was estimated on the basis of a power law scaling relationship with its population *P*_*U,φ*_^35^:

(2)AU,φ=αδPU,φβδ,forφ∈δ

where *α*_*δ*_ and *β*_*δ*_ are the proportionality coefficient and scaling factor for census division *δ* estimated by fitting log(*A*_*U*_) and log(P_U_) in 2000 according to equation (2). Strong linear relationships between log(*A*_*U*_) and log(P_U_) have been identified based on modern satellite imagery and census data^40–43^, and also observed in ancient settlements, for example, the Pre-Hispanic settlements in the Basin of Mexico^44^. Our hypothesis was that this scaling was stable and could be used to reconstruct historical settlement patterns. Empirical studies^45^ have revealed spatial variation of *β* values: 0.375 for England and Wales, 0.914 for Japan, 1.38 for China, and larger than 2/3 for US urbanized areas (population larger than 50,000). Spatial variation of *β* was incorporated here by developing such relationships for each census division, *δ*. However, the lack of corresponding *A*_*U*_ and *P*_*U*_ data for urban areas before 2000 precluded further evaluation of the temporal stationarity of *β*. Thus, the scaling relationships were considered stable for simplicity, with constant parameter values over time. The extent of rural areas (*A*_*R*_) within each county (*i*) was determined as the difference between the county total area (*A*_*T*_) and the aggregated extents of all urban areas in the county:

(3)AR=AT−∑AU,φ

Two simplifying assumptions were further made to delineate the extent of each urban area over time: 1) urbanization was monotonic, with historically established urban areas still extant in 2000; 2) urban areas developed outward from their center, so regions farthest from the center would urbanize last. The urban extents for each urban area in each decade were delineated by concentrically shrinking inward towards their center moving backward with time from 2000, with the remainder considered as rural areas.

### Influence coefficient calculation

Inhabitability, topographic suitability, and socio-economic desirability were the three major factors considered to influence human population distribution. The calculation method for the influence coefficient (*w*) of each factor for each pixel (*k*) is explained below. Note the influence coefficients for inhabitability (*w*_0*k*_) and topographic suitability (*w*_1*k*_) were steady over time, while the influence coefficient for socio-economic desirability (*w*_2*k*_) changed with the urbanization process.

#### Inhabitability

Inhabitable zones were defined here to exclude protected areas, waterbodies larger than 1 km^2^, and areas with elevation higher than 3500 m (Fig. 1). Protected areas were regarded as non-inhabitable if their status was defined as “Designated”, indicating legal or administrative decree, and if their public access was classified as “Restricted” or “Closed”. Note that protected areas are not constant during the time period of the study, and some populations were displaced in the creation of protected areas in the US. But the total number of such displaced persons is relatively low such that we expect the impact on our final results to be minor. Low-population census tracts were also treated as non-inhabitable areas. Preliminary analyses revealed that areal weighting resulted in overestimates for low population tracts. This simplification was found to reduce the overall model errors (see Table 1 for the specific cutoff tract population for each division). Inhabitable areas were assumed to be steady over time. The influence coefficient of inhabitability, *w*_0*k*_, for pixel *k* was set as zero for non-inhabitable areas and one for inhabitable areas.

#### Natural suitability

Elevation has a relatively larger variation and better resolution within counties compared to other natural factors, including temperature and precipitation, and plays an important role in influencing population distribution^46^. We aggregated this detailed elevation data to county level instead of census tract and county subdivision because the latter data are only recently available and are thus used here only for validation purposes. Although such aggregation masks variation within counties, especially for the western mountainous regions, we found that a valid relationship developed from a perspective of regionalization could generally reflect the topographic influence. We tested the relationship between county mean elevation and population density using linear, log, and logistic functions using the R statistical software. We chose elevation over slope to represent topographic suitability because we found that the former had a better linear relationship (larger R^2^) with population density

(4)ln(PDi)=mµzi+bµ,fori∈µ

where *i* and *μ* denote county and Region illustrated in Fig. 2, *PD*_*i*_ and *z*_*i*_ are population density and mean elevation for county *i*, and *b*_*μ*_ and *m*_*μ*_ are fitting parameters for Region *μ*.

We calculated the influence coefficient of elevation, *w*_1*k*_, for pixel *k* from the following relation

(5)w1k=emµzk,fork∈µ

where z_*k*_ is elevation for pixel *k*. Only inhabitable areas were considered when calculating population density. To minimize the number of model parameters, geographically adjacent census divisions and states with similar *m*_*μ*_ values from equation (4) were combined into seven regions (*μ*: R1 – R7, shown in Fig. 2), that upon integration still retained significant relationships between *z*_*i*_ and In (*PD*_*i*_). Two regions correspond to census divisions (R1 and R2), one includes only one state (R5, Florida), two comprise multiple contiguous states (R3 and R4), and two are composed of one census division plus multiple neighboring states (R6 and R7). No significant relationship was found for Colorado (R0, in Fig. 2), and thus population was not weighted by topographic suitability there. Significant negative linear relationships between *z*_*i*_ and In (*PD*_*i*_) were found for all regions except 100 m<*z*<300 m in R4 (Fig. 2). The presence of the city of Atlanta at approximately 300 m disrupted the general pattern that was found in the rest of the hot southeastern coastal plain. The slope, *m*, varied among regions, but all relationships were significant with *p*-value<0.001.

#### Socioeconomic desirability

We considered socioeconomic desirability for urban (*U*) and rural (*R*) areas separately. Urban population density decreases with increasing distance from the urban center. This trend has been described simply with an exponential decay model^47^, but with growing attention on fractal cities, an inverse power function has been used more recently^48–50^. Here, we used the inverse power function to describe how the influence coefficient of socioeconomic desirability *w*_2*k*_ for urban pixels (*k*∈*U*) changes across space.

(6)w2k=rkφ−λδ,fork∈U∧k∈δ

where *r*_*kφ*_ is radial distance from the center of urban area *φ* to the pixel *k*, and *λ*_*δ*_ is density gradient for division *δ*. We applied the following relation suggested by recent studies^51,52^ to link parameter *λ*_*δ*_ from socioeconomic desirability with the urban area-population scaling factor *β*_*δ*_ from equation (2)

(7)λδ=2−2βδ

Like *β*_*δ*_, *λ*_*δ*_ values were calculated for divisions and regarded as constant over time.

For rural areas, proximity to an urban center is advantageous for economic development. The influence coefficient of socioeconomic desirability w_2*k*_ for rural pixels (*k∈R*) was determined using a gravity model of market potential^53^

(8)w2k=∑φ=1NφPU,φrkφ2∑φ=1Nφ1rkφ2,fork∈R

where *N*_*φ*_ is the number of urban areas that are within the maximum urban influence distance in decade *t*, *D*_*t*_ (*r*_*kj*≤D*t*_), corresponding to daily per capita travel range which exponentially increased due to transport technology evolution from 30m in 1790 to 100 km in 2000 (ref. 54). The gravity model was adopted here, based on its wide application in reflecting the accessibility of urban markets^55–57^.

### Population mapping models

Five population distribution models of increasing complexity were developed, using the influence coefficients described above (normalized to range between 0 and 1). M1 simply allocated census county population homogeneously within counties. M2 separated urban and rural areas, and then homogeneously allocated urban and rural population within urban and rural areas, respectively. M3 excluded non-inhabitable areas, including waterbodies, protected areas, highlands, and low population census tracts. M4 extended Model 3 with the addition of topographic suitability. M5 added socio-economic desirability. From M3 to M5, the population distribution maps were obtained by multiplying the population raster by the normalized weighting grid, with each subsequent model contributing an additional influence coefficient to the previous model. The most complete model (M5) included all coefficients as shown in the following equation

(9)Pk=w0kw1ksw2kd∑w0kw1ksw2kdPZ,fork∈Z=(U,R)

where *Z* indicates urban (*U*) or rural (*R*) pixel, and the exponents *s* and *d* weight the relative importance of topographic suitability and socioeconomic desirability on population mapping. The latter two parameters were calibrated to obtain the highest mapping accuracy with values evaluated between 0.2 and 3 for each division (Tables 2 and 3). Equation 9 was implemented through Model Builder in ArcGIS to produce our historical population datasets. The models we used are publicly available and can be freely downloaded from figshare (Historical Population Models, Data Citation 1).

### Accuracy assessment

All five models, M1 - M5, were applied to map historical population from 1790 to 2010 (excluding 1960) (Data Citation 1). The best way to assess the accuracy of population distribution maps is to compare them with census data at a finer level than was used for the model input. While the county level input data were already the finest resolution that was available consistently throughout the temporal range of interest, higher resolution census tract data with full coverage of the conterminous US were available from 1990 to 2010, and these were used as a primary reference here. Note that the spatial resolution of our population products precluded validation for census tracts with area less than 1 km^2^, which represented 12.6% of the tracts and 9.7% of the total population for the conterminous US in 2010. We supplemented the tract analysis with reference data at a coarser level, county subdivision, from 1980 to 2010, to achieve a more comprehensive validation and also to assess the accuracy based on reference data at different geographic scales. We again used only county subdivisions with areas larger than 1 km^2^ (98.8% of the county subdivisions, and greater than 99% of the conterminous US population).

We generated gridded population data based on county level data and then aggregated population grids by census tracts and county subdivisions to compare them to census data at the corresponding level. We applied mean absolute relative error (MARE) to assess the overall performance of the 2000 population products for the conterminous US and each census division

(10)MARE=1Nc∑c=1Nc|Mc−OcOc|

where subscript *c* is reference data geographic unit (census tract or county subdivision), *N*_*c*_ is the number of evaluated units in the conterminous US or census division, *M*_*c*_ is modeled population aggregated for unit *c*, and *O*_*c*_ is observed population from census data.

The five models were assessed in terms of both accuracy and effectiveness. We used MARE to compare model accuracy, with lower MARE values indicating better model performance. Model effectiveness was determined from the importance of the added influence factors based on the magnitude of MARE reduction compared to the previous model. We made full use of the available fine-resolution census population data and assessed the accuracy of the model-generated historical population maps for the conterminous US based on census tract data from 1990 to 2010 and county subdivision data from 1980 to 2010. Furthermore, we assessed the accuracy of our generated historical urban extents for selected regions. There is currently no historical (urban) land use database for the USA from 1790 to present. However, the USGS LCI^36,37^ developed historical urban extents for two fast-growing regions: San Francisco/Sacramento and Baltimore/Washington DC. Those investigators mapped urban land use change and produced maps from 1900 to 1996 for San Francisco/Sacramento and from 1792 to 1992 for Baltimore/Washington DC. Therefore, the historical urban extents for these regions reconstructed in this study for the closest corresponding years were compared to the USGS LCI results to test the validity of the scaling relationships and the urban area delineation method. We used the relative overlap of urban pixels between our results and those from USGS LCI as an accuracy indicator. Note that the minimum population threshold used to define urban areas in the USGS LCI studies was 500 people, but we constrained our comparison to include only the areas with more than 2500 people, to be consistent with our threshold for urban areas.

Finally, we note that accuracy assessments for population products are commonly conducted using partial datasets due to the availability of higher resolution reference data. For example, Gaughan *et al.*^58^ used only four urban areas to validate population products for the entire mainland of China, and Sorichetta *et al.*^18^ used six countries for accuracy assessment of population products for 28 countries in Latin America and the Caribbean.

## Data Records

The reconstructed high-resolution historical human population dataset for the conterminous US from 1790 to 2010 (excluding 1960) are available on figshare, including five sets of population products derived from M1 to M5 (Historical population dataset for the conterminous US, Data Citation 1). The data can be downloaded in Esri grid format for each decade at a resolution of 1 km, with the values representing the human population per pixel cell.

## Technical Validation

### Population mapping illustration

**Figure 3:**
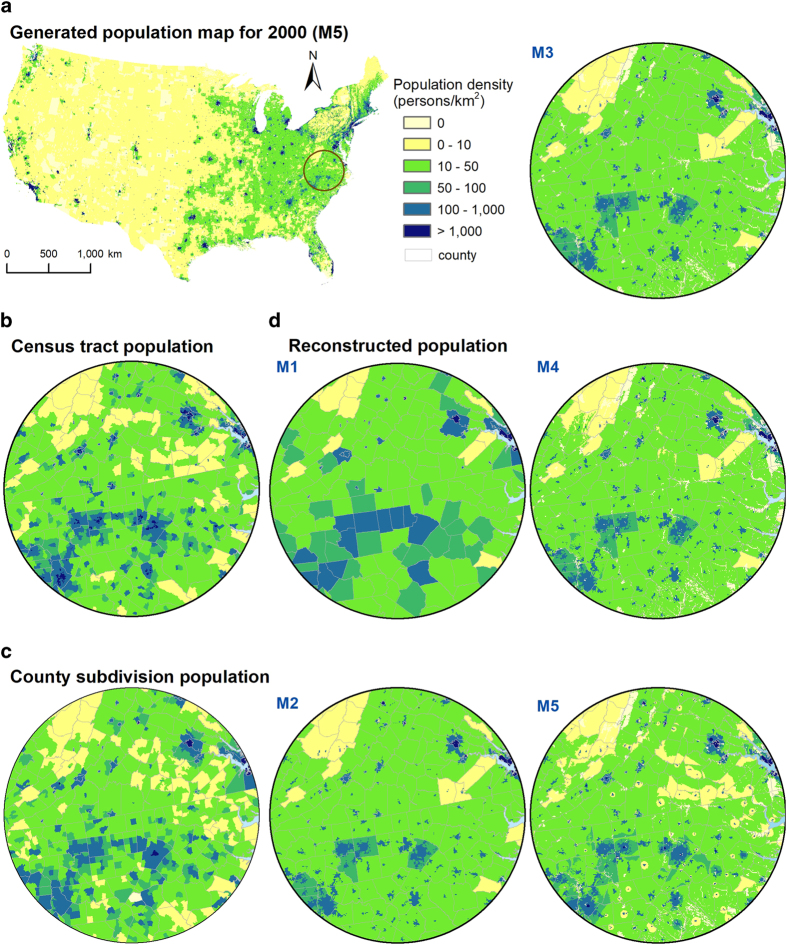
Comparison of measured and modeled population maps for year 2000. Population map generated by M5 for the conterminous US (**a**), census tract population (**b**), county subdivision population (**c**), and the five model outputs (M1 - M5) (**d**) in the South Atlantic region.

The spatial details generated by M1 to M5 are illustrated in Fig. 3, with a focus on the northern part of the South Atlantic division (D5), which exhibits significant topographical variation from the coastal plain to the Appalachian Plateau, and includes many cities of different sizes. The census tract (Fig. 3b) and county subdivision data (Fig. 3c) are compared to all five model outputs (Fig. 3d). M1 and M2 produced homogeneous populations within counties, and urban and rural areas, respectively. M3 also produced homogeneous populations within urban and rural areas, but had more low population pixels due to the exclusion of non-inhabitable areas. M4 and M5 generated more spatial details within urban and rural areas for each county. With input from both topographic suitability and socio-economic desirability, M5 produced more detailed information consistent with the census tract population distribution, not only for the less densely populated zone in the northeast but also the high population regions in the southwest.

### Population mapping model accuracy

**Figure 4:**
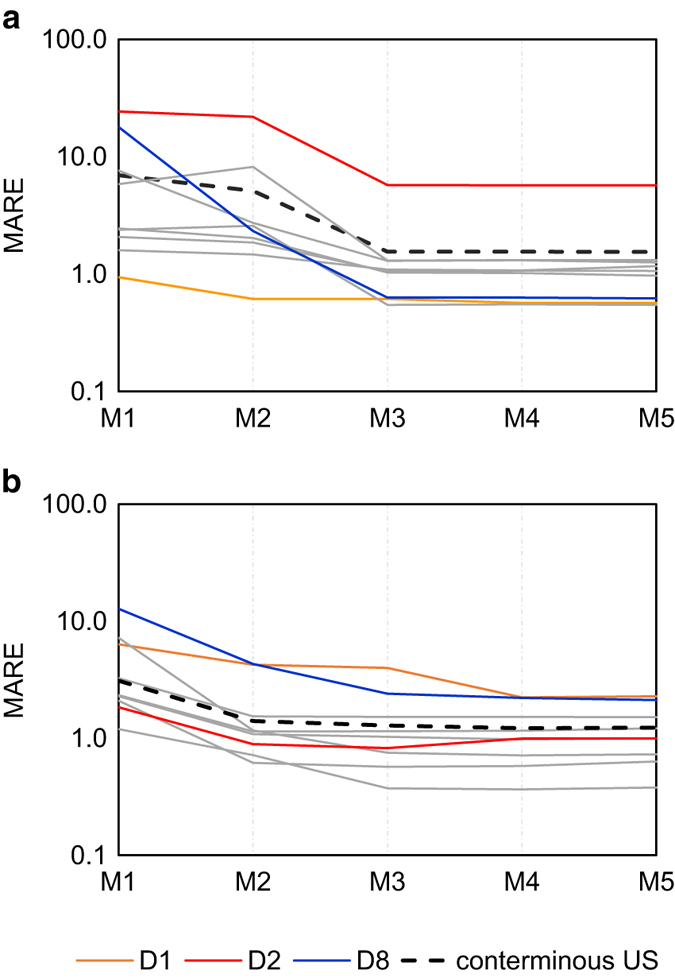
Population mapping model performance comparison. (**a**) comparison based on census tract population data; (**b**) comparison based on county subdivision population data. Mean absolute relative error (MARE), on a log scale, for most census divisions (grey lines) showed similar trends in improved model performance with increasing model complexity. The orange, red, and blue lines indicate census divisions D1, D2, and D8 for comparison. The dashed line indicates model performance for the entire conterminous US.

Census tract population data was considered as the primary reference for validation due to its higher resolution, with mean area and population of 120 km^2^ and 4310 persons in 2000. Based on our validation using census tract population for year 2000 (Fig. 4a) for the conterminous US, accuracy increased with model complexity from M1 to M5, with MARE decreasing from 6.96 (M1) to 1.54 (M5), indicating M5 as the most accurate model. By census division (*δ*: D1 - D9), model accuracy showed similar improving trends from M1 to M5, except M2 for D4 and D9. The model performed worst for D2, however, this division also showed improved performance with increased model complexity.

Validation using county subdivision population data (mean area and population of 221 km^2^ and 7932 persons) resulted in similar overall results, but with higher accuracy, indicated by smaller MARE values for Models 1 to 5 (Fig. 4b). The MARE decreased from M1 (3.11) to M4 (1.22), then increased marginally (1.23) for M5, showing M4 as the most accurate model. Similar trends were found for most divisions, except D2 and D5, with M3 as the most accurate model, and also D3, where model accuracy decreased from M2 (1.14) to M5 (1.22). Note that small or no differences in MARE were found between the most accurate models and M5 for these divisions. D1 and D8 presented worse model performance than the other divisions.

### Population mapping model effectiveness

The improved accuracy found above was achieved by the increased complexity from M1 to M5. Models 1 to 5 increased in complexity by adding additional input data. M1 required only one data set (county total population, *P*_*T*_), M2 required the addition of urban population (*P*_*U*_) and extent of urban areas (*A*_*U*_) for a total of 3 variables, M3 added inhabitable area for 4 variables, M4 also included topography and its relative importance (*s*) for 6 variables, and M5 added socioeconomic desirability and its relative importance (*d*), culminating in 8 variables.

Census-tract-based validation for the conterminous US (Table 4) showed that M3 and 2 resulted in the largest reduction in MARE compared to their next lower-complexity models: 3.58 and 1.53, respectively, revealing the importance of separating urban and rural areas as well as excluding non-inhabitable areas. In contrast, M4 and M5 resulted in only small reductions in MARE. Also, the additional factors in each model showed varied effects among the nine divisions. From M1 to M2, the largest MARE reduction (15.67) was found for the Mountain Division (D8), demonstrating the importance of differentiating urban and rural areas where tract areas have both high mean and high variance. From M2 to M3, the Middle Atlantic Division (D2) showed the largest MARE reduction (16.12), indicating the significant effect of excluding un-inhabitable areas where population density is high. The MARE reductions from M3 to M4 and M4 to M5 were small in all regions. Overall, the magnitude of MARE reduction decreased as the model complexity increased from M1 to M5 (Table 4). Although the most complex model M5 had the lowest MARE, the model effectiveness^59^ considering both model accuracy and model complexity decreased from M3 to M5. We thus considered M3 as the most efficient model.

### Urban extent dynamics

**Figure 5:**
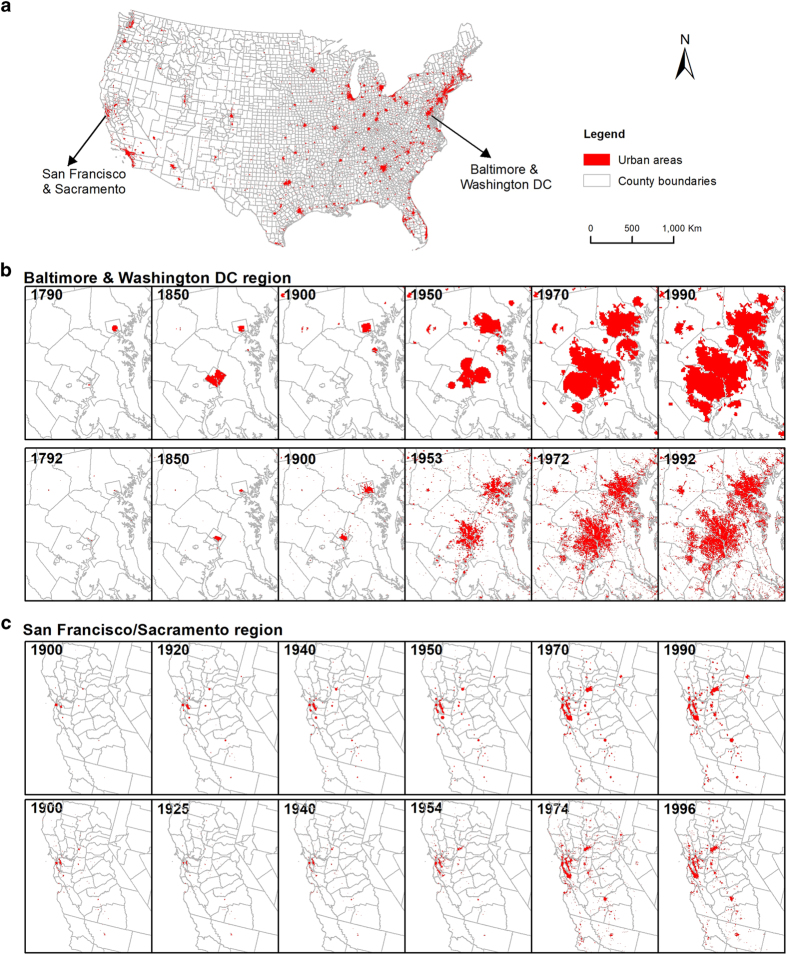
Comparison of reconstructed historical urban extents. Urban area distribution in 2000 (**a**), Baltimore-Washington DC from 1790 to 1790 from this study (top panel) and reference^37^ (bottom) (**b**), and San Francisco/Sacramento from 1900 to 1990 from this study (top) and reference^36^ (bottom) (**c**).

The historical urban areas were reconstructed based on the oldest available urban area boundaries (2000) using the scaling relationship between urban area and population. We illustrated the urban extents for Baltimore/Washington DC and San Francisco/Sacramento regions from our reconstructed results for the closest corresponding years and the USGS LCI results in Fig. 5. Generally, our reconstructed urban areas matched well with the USGS products especially since the 1950s, albeit with some deviation in the location centers from which the urban areas developed outward in the early decades. For Baltimore/Washington DC, the fraction of USGS LCI urban pixels that were overlapped by our model were 0.88, 0.84, 0.73, 0.70, and 0.92 for 1990, 1970, 1950, 1900, and 1850. For San Francisco/Sacramento region, the fractions were 0.78, 0.82, 0.73, 0.65, 0.50, and 0.62 for 1990 to 1900 (Fig. 5). Our simplified reconstruction tended to overestimate urban extents in both regions, and the lower population thresholds used to define urban areas for the USGS LCI studies resulted in more scattered small urban areas than in our results. The assessment of the historical urban areas in two fast-growing regions showed that our reconstructed urban extents reflect the general dynamic pattern of urban areas compared to the USGS LCI reference data.

### Validation of generated historical population

Historical population distribution was reconstructed for the conterminous US from 1790 to 2010 (excluding 1960). Validation is only possible beginning with the available data. For census-tract-based validation (1990-2010), since Models 3 to 5 were calibrated based on 2000 population data, their accuracy for 1990 was unsurprisingly lower than the 2000 output. For example, for M5 MARE values were 2.1 and 1.5 for the conterminous US in 1990 and 2000, respectively (Table 5). Using the five models to project forward from 2000 to 2010 for the conterminous US also had higher MARE values except M2. However, based on county subdivision validation (1980–2010), our population products showed high accuracy for both backward projection from 2000 (1990 and 1980) and forward projection (2010), with similar accuracy as the 2000 products. Our population products prior to 1980 could be validated if census tract, county subdivision, or other more detailed population data become available.

## Usage Notes

The dataset generated here provided ready-to-use historical population maps in the conterminous US from 1790 to 2010 at the resolution of 1 km. Our study showed the validity of applying scaling relationships between urban area and urban population to reconstruct historical urban areas, and the effectiveness of modeling spatio-temporal population distributions using existing data. Our backward projection was not just based on current population information but historical population data obtained from NHGIS, which provided accurate information about human settlement and population density at county level and served as a foundation to further disaggregation of population within administrative units. The high resolution of the input census population data, the separation of urban and rural extents, and use of inhabitability, elevation, and socioeconomic desirability influencing factors all contributed to the good accuracy of the final products.

Our final output included five sets of historical population products from 1790 to 2010 reconstructed based on models M1 to M5. Generally, model accuracy improved with increasing complexity. According to the census-tract-based validation, we proposed M5 as the most accurate and M3 as the most efficient, while the county-subdivision-based validation suggested M4 as the most accurate and M2 as the most efficient, largely because the larger size of county subdivisions masked the effect of additional factors included in more complex models. Therefore, we suggest that users consider their study units when selecting the model products. Also, we suggest applying the efficient models when transferring our approach to other regions, and adopting the most accurate models when directly applying our model-generated population products. Additionally, considering the varied model performance among the divisions, we caution users to choose the most appropriate products based on their specific study regions.

When compared to the large task of modeling human population at a continental scale over a time period of hundreds of years, our models include only a small number of parameters, and we thus consider all five models M1 to M5 as parsimonious. Our models used no more than three weighting coefficients, partly determined by the availability of historical geospatial data, while, for comparison, 10 weighting coefficients were used in LandScan^16^. Similar to our method, Landscan^16^ and population modeling for Asia^19^ and Africa^20,21^ also applied multiple ancillary variables and allocated population using associated weights. However, land cover data, which were not available over the long time scales of interest in this study, were the major input for their modeling. Landscan did not provide details on their modeling methods^19^. The population mapping influence coefficients previously developed^19–21^ were based on fixed values of population density for specific land covers, while those we developed as continuous functions of elevation, urban proximity, and market potential (equations (5),(6) and (8)) supported more variation. Further, our reconstruction was based on the same model structure with consideration of the dynamic trends of the model input parameters over time, including the number of urban areas and urban population, increasing urban influence distance over time, and changes of density gradients within urban areas.

Natural factors other than altitude, including temperature and precipitation, were excluded because of their relatively small variation within most counties. However, altitude was found to be an important factor to account for intra-county natural suitability variability. We further emphasize that our validated population products used the same method for the entire conterminous US, with no significant accuracy differences in the East and West. Another important factor that could be considered more explicitly in extensions to this work is transport networks, given their significant role in influencing human population distribution. Transport networks have evolved over time, shifting from rivers and canals, to roads and railroads, and then to air transport^54^, but there are no currently available comprehensive historical databases of transportation networks (rail lines, roads), precluding their direct inclusion in our models. However, the effect of the transport technology evolution was indirectly reflected in our models through the growth of the daily travel range in the gravity model of market potential. The availability of temporally dynamic data limited the selection of influencing factors. We aimed to apply consistent techniques over time to make the final products appropriate for dynamic analysis.

Our historical population products were generated and validated based on census population data. This data-based approach was therefore subject to the limitation of the currently available census data. First, we did not consider American Indians in our population distribution reconstruction since census data did not include American Indians prior to 1900. It is still a challenge to collect and confirm population numbers for American Indians due to the sparse and inconsistent record. Second, urban areas are defined based on a population threshold of 2500, however, areas with less than 2500 people in the past could have held a role similar to modern urban areas. Our reconstruction thus might underestimate the attractiveness of smaller urban areas. Third, lack of high resolution historical data constrained a comprehensive validation dating back to 1790. Our historical population products prior to 1980 can be validated when more detailed census data become available. Despite these limitations, the population data from US Census Bureau are the best currently available source for data-driven analyses.

**Figure 6:**
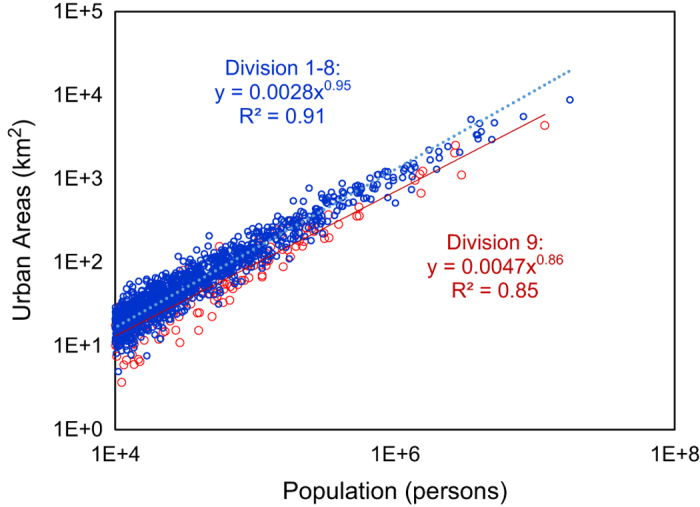
Scaling relationship between urban population and urban area, 2000.

We applied the following major assumptions in this study: Monotonic urbanization and homogeneously outward expansion, constant *β*, and steady inhabitable areas. The first two assumptions were used to reconstruct historical urban areas and the third was applied to determine the influence coefficient of inhabitability. While individual urban development patterns may often deviate from the first assumption (such as linear expansions along rivers or roads), simplifications were necessary to develop efficient models that could be feasibly applied to the large spatial and temporal scale of our study. This assumption could lead to varied impacts on model accuracy for specific cities with different development patterns. However, we found that the dynamic urban extents we generated reflect the general pattern of urban areas based on comparison with measured data available for two fast-growing regions, Baltimore-Washington DC dating back to 1792 and San Francisco/Sacramento dating to 1900 (Fig. 5). For the second assumption of constant *β*, currently there is no consistent opinion on how *β* may change over time. Theoretical considerations from a geometric perspective suggested *β*=2/3, with dimensions of 3 for population and 2 for area^35^, although there is some controversy on the dimension of population. Temporal analyses based on a model of settlement structure and social networks^44^ provided support for the dynamic nature of *β*: between 2/3 for unstructured settlements and 5/6 for larger and denser settlements with infrastructure networks. However, empirical analysis discovered little variation in exponents for Taiwan at different development phases^41^. Here, we found *β*=0.95 for all divisions except Pacific (D9) with *β*=0.86 (Fig. 6) based on current urban data, however, a lack of historical urban areas data could not support a dynamic analysis on this exponent and we assumed it to be constant. A smaller *β* for unstructured settlements^44^ would suggest smaller sizes of urban areas and thus a higher population density in earlier times, which might also explain our overestimation of historical urban extents. Regarding our third assumption, the total inhabitable area may have expanded with increasing human settlement pressure and advancing technological development. For example, widespread drainage converted wetlands to croplands and settlements^60^. Thus, our model may overestimate historical inhabitable areas and thus underestimate population density in previous decades. However, data availability limitations necessitated simplifying assumptions, which result in inevitable modelling limitations. Future work could improve these models as more data become available.

The spatially explicit historical population data generated here could facilitate advancing our understanding of coupled human and natural systems. With the arrival of the Anthropocene, the scope, intensity, and rate of changes in human-nature interactions have increased dramatically^3^. It is thus becoming increasingly important to understand the dynamics of human-nature interactions at time scales of decades to centuries^8^. For example, from the perspective of water resources, several studies have quantified the geographic relationship between human settlements and rivers^61,62^, however, it remains elusive how such relationships have changed over time. Our population products could help evaluate dynamic human-nature relationships.

## Additional information

**How to cite this article:** Fang, Y. & Jawitz, J. W. High-Resolution Reconstruction of the United States Human Population Distribution, 1790-2010. *Sci. Data* 5:180067 doi: 10.1038/sdata.2018.67 (2018).

**Publisher’s note:** Springer Nature remains neutral with regard to jurisdictional claims in published maps and institutional affiliations.

## Supplementary Material



## Figures and Tables

**Table 1:** Determination of cutoff population below which low-population census tracts were treated as non-inhabitable areas for each division.

**Division**	**MARE, when cutoff population=**					**cutoff population (persons)**
	**1000 persons**	**1500 persons**	**2000 persons**	**2500 persons**	**3000 persons**	
Conterminous US	1.19	1.10	1.07	1.15	1.30	
D1	0.59	0.60	0.61	0.64	0.70	1000
D2	4.21	3.97	3.71	3.97	4.55	2000
D3	0.94	0.64	0.53	0.58	0.67	2000
D4	0.44	0.44	0.46	0.54	0.66	1500
D5	0.95	0.72	0.76	0.85	0.99	1500
D6	0.41	0.41	0.45	0.49	0.57	1000
D7	0.54	0.53	0.55	0.60	0.66	1500
D8	0.43	0.44	0.44	0.45	0.49	1500
D9	1.16	1.51	1.51	1.55	1.63	1000

**Table 2:** Determination of the topographical suitability factor, s, for each division.

**Division**	**MARE, when s=**														**s**
	**0.2**	**0.4**	**0.6**	**0.8**	**1.0**	**1.4**	**1.5**	**1.8**	**2.0**	**2.2**	**2.4**	**2.6**	**2.8**	**3.0**	
Conterminous US	1.01	1.01	1.01	1.01	1.01	1.01	1.01	1.02	1.02	1.03	1.03	1.04	1.05	1.05	
D1	0.58	0.56	0.55	0.55	0.55	0.55	0.55	0.56	0.57	0.58	0.59	0.60	0.61	0.62	1.00
D2	3.74	3.69	3.64	3.60	3.57	3.52	3.49	3.45	3.43	3.41	3.39	3.37	3.36	3.35	3.00
D3	0.52	0.52	0.52	0.52	0.52	0.52	0.52	0.52	0.52	0.52	0.52	0.52	0.52	0.52	1.00
D4	0.45	0.45	0.45	0.45	0.45	0.45	0.45	0.45	0.46	0.46	0.46	0.46	0.46	0.46	1.00
D5	0.73	0.74	0.75	0.76	0.78	0.80	0.82	0.84	0.86	0.88	0.89	0.91	0.93	0.95	0.20
D6	0.41	0.41	0.41	0.41	0.41	0.41	0.41	0.41	0.41	0.41	0.41	0.42	0.42	0.42	1.00
D7	0.53	0.52	0.52	0.52	0.52	0.52	0.52	0.52	0.52	0.52	0.52	0.52	0.52	0.52	1.00
D8	0.68	0.68	0.68	0.68	0.68	0.68	0.69	0.69	0.70	0.70	0.71	0.71	0.72	0.72	1.00
D9	1.17	1.19	1.20	1.22	1.24	1.27	1.29	1.32	1.35	1.37	1.39	1.41	1.44	1.46	0.20

**Table 3:** Determination of the socio-economic desirability factor, *d*, for each division.

**Division**	**MARE, when** ***d*****=**															***d***
	**0.02**	**0.04**	**0.06**	**0.08**	**0.10**	**0.20**	**0.40**	**0.60**	**0.80**	**1.00**	**1.20**	**1.40**	**1.60**	**1.80**	**2.00**	
Conterminous US	0.966	0.966	0.966	0.967	0.967	0.968	0.972	0.979	0.987	0.998	1.010	1.025	1.042	1.061	1.081	
D1	0.546	0.546	0.545	0.545	0.545	0.545	0.547	0.551	0.556	0.563	0.572	0.581	0.592	0.603	0.616	0.20
D2	3.353	3.359	3.365	3.371	3.378	3.410	3.479	3.552	3.632	3.717	3.809	3.907	4.011	4.123	4.241	0.02
D3	0.518	0.517	0.517	0.517	0.516	0.515	0.513	0.513	0.515	0.519	0.525	0.532	0.540	0.549	0.560	0.40
D4	0.452	0.451	0.451	0.451	0.451	0.449	0.448	0.448	0.449	0.452	0.456	0.462	0.468	0.476	0.485	0.60
D5	0.729	0.728	0.727	0.726	0.725	0.720	0.713	0.707	0.702	0.700	0.699	0.700	0.702	0.706	0.710	1.00
D6	0.408	0.408	0.407	0.407	0.407	0.407	0.407	0.409	0.413	0.418	0.425	0.433	0.443	0.454	0.466	0.20
D7	0.522	0.521	0.521	0.520	0.520	0.518	0.515	0.513	0.514	0.517	0.521	0.527	0.534	0.542	0.551	0.60
D8	0.681	0.680	0.680	0.680	0.680	0.679	0.678	0.679	0.682	0.687	0.692	0.700	0.709	0.719	0.729	0.40
D9	1.169	1.168	1.166	1.165	1.164	1.158	1.147	1.137	1.129	1.122	1.117	1.113	1.111	1.110	1.110	1.80

**Table 4:** Model effectiveness assessment results by Division for 2000 population products.

**Division**	**census tract MARE reduction**				**county subdivision MARE reduction**			
	**M1-M2**	**M2-M3**	**M3-M4**	**M4-M5**	**M1-M2**	**M2-M3**	**M3-M4**	**M4-M5**
conterminous US	1.83	3.58	0.00	0.01	1.71	0.12	0.07	-0.02
D1	0.33	0.00	0.05	0.00	2.10	0.25	1.76	-0.04
D2	2.40	16.12	0.03	-0.01	0.95	0.07	−0.17	0.00
D3	0.22	0.81	−0.01	0.00	1.20	−0.01	−0.01	−0.06
D4	−0.19	2.03	0.00	0.00	1.72	0.02	0.00	0.01
D5	0.41	1.00	0.01	0.05	1.47	0.05	−0.01	−0.05
D6	0.12	0.38	0.02	−0.10	0.48	0.34	0.01	−0.01
D7	4.91	1.44	−0.01	0.05	1.20	0.06	0.05	−0.02
D8	15.67	1.70	0.00	0.01	8.50	1.92	0.19	0.09
D9	−2.36	6.89	−0.02	0.00	6.06	0.42	0.04	−0.01

**Table 5:** Model accuracy assessment results by model based on census tract population (1990–2010) and county subdivision population (1980–2010).

**Model**	**census tract MARE**				**county subdivision MARE**		
	**1990**	**2000**	**2010**	**1980**	**1990**	**2000**	**2010**
M1	4.29	6.96	8.02	2.96	3.03	3.11	5.20
M2	2.94	5.13	4.94	1.46	1.45	1.40	1.42
M3	2.11	1.55	3.56	1.34	1.32	1.29	1.29
M4	2.19	1.55	3.56	1.26	1.20	1.22	1.21
M5	2.13	1.54	3.55	1.27	1.18	1.23	1.24
